# Correction

**DOI:** 10.1080/22221751.2026.2678031

**Published:** 2026-05-28

**Article title**: Single-cell sequencing of peripheral blood mononuclear cells reveals immune landscape of monkeypox patients with HIV

**Authors**: Liu, Yamin; Liu, Xinhua; Wang, Jingjing; Xie, Ying; Guo, Jing; Liu, Zhiqiang; Li, Ying; Jiang, Bei & Wang, Jingya.

**Journal**: *Emerging Microbes & Infections*

**Bibliometrics**: Volume 14, Issue 1

**DOI**: https://doi.org/10.1080/22221751.2025.2459136

The citation mistake in the single-cell sequencing procedure of the Methods section has been corrected, and the authors have updated and cited the relevant literature. In the corrected version, the newly added relevant reference is listed as reference 64.

